# Mr. Smith's Case of Extirpation of Diseased Tonsils

**Published:** 1813-04

**Authors:** Robert Smith

**Affiliations:** Member of the Royal College of Surgeons, and Surgeon to the Chelsea and Brompton Dispensary. Sloane Street


THE
### Medical and Physical Journal.

4 OF VOL. XXIX.] APRIL, 1813. [NO. 170.

*To the Editors of the Medical and Physical Journal.*

GENTLEMEN,

I DO not request you to insert the following case as novel or curious, either in itself or the mode of treatment; but with a view of inducing others more readily to undertake a simple operation, for the cure of a most distressing malady.

October 19, 1812, John Windle, residing in New Court, near Sloane Street, a boy about ten years of age, and of an unhealthy appearance, was brought to me at the Chelsea and Brompton Dispensary, in consequence of a considerable enlargement of both tonsils, which had existed from his infancy, and gradually increased so as now to threaten suffocation. To this cause, together with a defect in the palate, may be attributed his inability to speak till he was five years old, and even now his articulation is very indistinct. About three years and a half since, the distressing sensations in his throat became so urgent as to induce his parents to apply to different physicians, and afterwards to several surgeons, of eminence. Various remedies, the nature of which are not known, were prescribed internally and externally, with little or no advantage. He was next taken to one of the hospitals of this metropolis, where the tonsils were several times punctured with temporary relief; and blisters, which had formerly been applied and kept open, were again had recourse to without benefit.

The complaints still increasing, and despairing of obtaining relief from any regular practitioner, his parents were persuaded to apply to several empirics, who pretended to cure similar affections, and from whom they procured, at considerable expense, gargles and other nostrums, which were persisted in some time with no advantage. Unable to purchase any further] advice, and having derived so little benefit from what they had already obtained, they were induced to consider the state of the boy as nearly hopeless, and to abandon all applications. In the mean time the disease increased to such a degree, that he was frequently unable to swallow a sufficient quantity of food to satisfy his hunger. His nights were peculiarly distressing, the breathing often becoming so laborious, that, to prevent suffocation, his father or mother were obliged to watch him, and occasionally to raise him up in bed, as the only means of affording relief. On examination of the throat, I found both tonsils were so much enlarged as to be nearly in contact with each other, leaving merely a small fissure into the pharynx. They were of their natural color, but somewhat firmer than usual, and the surface of each was marked by several indentations, which seemed to have been occasioned by the punctures. As some decisive means of relief were now absolutely necessary to preserve the boy's life, and the removal of the tonsils the only effectual mode of obtaining it, I proposed their extirpation by ligature, which was readily acceded to by the patient and his father. On account of the great size of the tonsils, and the inflammation and inconvenience which might be expected to follow the operation, I determined on first removing the right, which was the largest, but knew of no instrument by which I could readily pass a ligature; I therefore made use of a strong flat probe, curved in such a manner as to pass round the base of the tumor, having a groove on its convex side, with a small eye as near as possible to its extremity, while the opposite one was bent so as to form a kind of handle.

October 23.—I passed a strong ligature in the following manner: having fastened the centre of it to the eye in the extremity of the probe by a single thread, rather loosely, so that it might be readily divided, I then held one end firmly in my left hand, and with the fore-finger depressed the tongue; while in the right I held the probe, with the ligature resting in its groove, which I passed low down in the throat, with the concave side applied to the tonsil; and, then pressing it backward and upwards, keeping the end of it close to the basis of the tonsil, brought it over the superior part, and divided the thread which fastened the ligature to the probe, then tied it moderately tight, and cut it off nearly close to the knot.

The tumor looked dark, and the boy immediately complained of some pain, and a great flow of saliva took place. I desired the patient to be kept perfectly quiet, and merely to take a little broth, gruel, or milk.

24th.—The boy had passed a restless night, although the pain had nearly ceased about an hour after the operation; but he suffered considerable inconvenience from the constant discharge of saliva. The tumor appeared dark, and nearly insensible to the touch, and the ligature had cut through the anterior part of it about the eighth of an inch. I directed a common gargle to be used frequently.

25th.—The tumor was less, and the surface sloughy; he had had passed an indifferent night, owing to the constant flow of saliva.

26th.—I removed the ligature, and passed another from above in the manner already described, and tied it tighter than the first; the tumor looked very dark, and the boy complained of considerable pain for two or three hours.

27th.—Part of the tumor had sloughed off, and the remainder had assumed its natural color. The boy had passed a better night, and expressed himself much relieved from the sense of suffocation; the flow of saliva was still, however, very troublesome.

31st.—The ligature having become loose, I passed another through it, above the knot, by means of a small-eyed probe, bent at its extremity; and, having fastened it, cut the former one beneath; then, taking hold of the lower end with a pair of forceps, drew it away, and at the same time brought the new ligature round the basis of the tonsil, which I tied tight. The tumor became, as before, dark, the pain was considerable, and the salivary discharge increased.

November 4th.—The tumor was less, but had recovered its red appearance, in consequence of the slough having been thrown off; the ligature had produced ulceration, and was becoming loose.

5th.—I drew another ligature round in a similar manner to the last, and tied it with considerable force, using the tonsil iron. The usual symptoms followed.

6th.—The tonsil was sloughy, and in the evening the boy was sensible of its having sunk in his throat; discharge of saliva much the same.

7th.—He had passed an indifferent night; the tumor still appeared dark, and, being now moveable in the throat, I passed a dissecting hook into it, and cut it off with a pair of scissars, at the place where the ligatures had been tied; no hemorrhage ensued, and the boy expressed himself greatly relieved. The tumor, when removed, was about the size of a small walnut; of course much less than before a ligature was passed round it.

8th.—The boy, to use his father's expression, "had never slept so well in his life," not having awoke from eight in the evening till eight the following morning. The right side of the throat was perfectly smooth, and free from any enlargement; the discharge of saliva had ceased; his breathing was relieved, and speech improved.

9th.—As the left tonsil filled nearly half of the throat, though not so large as the right, I also advised its removal, and passed a ligature round, which I drew very tight with the tonsil iron. The boy complained of great pain for a

 considerable considerable time, followed by the symptoms before enumerated. I passed another ligature on the 14th, 18th, and 21st, and in the evening of the 22d I removed the tumor with a pair of scissars; a very trifling hemorrhage followed.

23d.—There was a little slough, which was thrown off in a day or two.

26th.—He was quite well. He took no medicine, except occasionally an aperient.

February 12, 1813.—The boy is perfectly well; sleeps all night; no difficulty in breathing or swallowing: he speaks more distinctly, and his general health is much improved.

I am, Gentlemen,

Your most obedient Servant,

*Sloane Street,*
*Feb.* 13, 1813.

ROBERT SMITH,
Member of the Royal College of Surgeons, and Surgeon to the Chelsea and Brompton Dispensary.

